# Microbial deactivation properties of generation 2 and 4 poly(amidoamine) dendrimers on common bacteria found in the aqueous environment

**DOI:** 10.1186/2047-2994-4-S1-P35

**Published:** 2015-06-16

**Authors:** A Maleki, M Ahmadi Jebelli, B Hayati, H Daraei, F Gharibi

**Affiliations:** 1 Kurdistan Environmental Health Research Center, Kurdistan University of Medical Sciences, Sanandaj, Iran, Islamic Republic Of

## Introduction

The antibacterial properties of dendrimers, make them a good candidate, as a future alternative disinfectant, for water and wastewater treatment with minimal side effects. Therefore, this study was performed for evaluation of antibacterial effect of generation-2 and 4 poly(amidoamine) dendrimer (PAMAM) on the indicator bacteria found in the water resources.

## Methods

Using the differential biochemical tests, bacteria were isolated and identified from water resources. The minimum inhibitory concentration (MIC) and minimum bactericidal concentration (MBC) against gram-positive and gram-negative bacteria were calculated. Standard discs were prepared by different concentrations of dendrimer (0.5 to 500 μg/ml) and evaluated through the disc agar diffusion method on Muller-Hinton agar plates. Finally, the inhibition zone diameter was measured.

## Results

Main isolated bacteria from water resource were *Escherichia coli*, *Pseudomonas aeruginosa*, *klebsiella oxytoca*, *Bacillus subtilis*, and *staphylococcus aureus*. The results showed that the MIC and MBC for each of isolated bacteria were the same for both generations and were as follows: *Escherichia coli* 1250 and 2500 μg/ml; *klebsiella oxytoca* 500 and 1250 μg/ml; *staphylococcus aureus* 1 and 5 μg/ml and *Bacillus subtilis* 2.5 and 5 μg/ml. No MIC and MBC were observed on *Pseudomonas aeruginosa*, respectively. Also it was found that PAMAM dendrimer was more potent towards the gram positive bacteria than the gram negative bacteria. Although amino terminated G4 PAMAM dendrimers have a more functional groups, but no significant differences were observed in antimicrobial activity than G2 PAMAM dendrimers.

## Conclusion

It can be concluded that G2 and G4 PAMAM dendrimer with amine terminations exhibited a positive impact on the removal of dominant isolates strains. It is therefore possible that in the future it could be used as an effective material for water disinfection.

## Disclosure of interest

None declared.

